# Cervical HPV infection in Yueyang, China: a cross-sectional study of 125,604 women from 2019 to 2022

**DOI:** 10.3389/fpubh.2023.1210253

**Published:** 2023-08-03

**Authors:** Jinfeng Hou, Min Zeng, Chongmei Liu, Bochao Xie, Yu Li, Longyun Wu, Long Zhu, Manqiu Li, Zhihui Zhang, Xiaoyun Zhang, Yangqing Ge

**Affiliations:** ^1^Department of Gynecology, Yueyang People’s Hospital, Hunan Normal University, Yueyang, China; ^2^Department of Pathology, Yueyang Central Hospital, Yueyang, China; ^3^Department of Pathology, Huarong County People’s Hospital of Hunan Province, Yueyang, China; ^4^Department of Pathology, Pingjiang People’s Hospital of Hunan Province, Yueyang, China; ^5^Department of Pathology, Linxiang People’s Hospital of Hunan Province, Yueyang, China

**Keywords:** human papillomavirus, genotypes, prevalence, epidemiology, cervical lesions

## Abstract

**Objective:**

Human papillomavirus (HPV) infection is currently the main cause of cervical cancer and precancerous lesions in women. The aim of this study was to investigate the epidemiological characteristics of HPV genotypes among women in Yueyang city and to provide a basis for the prevention and treatment of cervical cancer in this city.

**Methods:**

A cross-sectional study was conducted on 125,604 women who had received treatment from eight hospitals in Yueyang city from September 2019 to September 2022. Analysis of the prevalence of HPV in patients.

**Results:**

The prevalence of HPV was 20.5% (95%CI: 20.2–20.7%), of which the high-risk type (HR-HPV) accounted for 17.5% (95%CI: 17.3–17.7%) and the low-risk type (LR-HPV) accounted for 5.0% (95%CI: 4.9–5.1%). Among the HR-HPV subtypes, the top five in prevalence, from the highest to the lowest, were HPV52 (5.1%), HPV16(2.7%), HPV58 (2.6%), HPV53 (2.4%), and HPV51 (1.7%). The main LR-HPV infection types were HPV81 (2,676 cases, OR = 2.1%; 95%CI, 2.0–2.1%). Among the infected patients, 19,203 cases (OR = 74.3%; 95%CI, 73.8–74.9%) had a single subtype, 4,673 cases (OR = 18.1%; 95%CI, 17.6–18.6%) had two subtypes, and 1957 cases (OR = 7.6%; 95%CI, 7.3–7.9%) had three or more subtypes. HPV prevalence is highest among women <25 years, 55–64 years and ≥ 65 years of age.

**Conclusion:**

The prevalence of HPV in women in Yueyang city was 20.5%, with HR-HPV being dominant. As women aged <25 years, 55–64 years, and ≥ 65 years are at a relatively higher risk, more attention should be paid to them for prevention and control of HPV infections.

## Introduction

Cervical cancer is one of the most common cancers in women. In 2020, 604,127 new cases and 341,831 deaths from cervical cancer were reported worldwide (1). In China, 109,741 new cases and 59,060 deaths from cervical cancer were reported in 2020 (2); therefore, cervical cancer remains a serious health threat to women in China. Human papillomavirus (HPV) is a circular, non-enveloped deoxyribonucleic acid (DNA) virus that can cause various lesions on the skin and mucous membranes. HPV infection is a common sexually transmitted disease (3), and approximately 75% of sexually active people become infected with this virus (4). Although most HPV infections are short-lived and do not seriously cause disease, some of these infections that last for several years may have a high potential to lead to malignancies (5). Persistent high-risk HPV (HR-HPV) infection is a critical step in cervical carcinogenesis (6, 7). Cervical cancer can be prevented, detected, and treated early. As such, early prevention has become the focus of cervical cancer prevention. At present, three types of vaccines have been licensed internationally for HPV infections. Vaccination has been effective in preventing cervical cancer in developed countries. However, only a minority of women in China have been vaccinated (8). Many studies suggest that the prevalence and genotype distribution of HPV vary by climate, region, ethnicity, and country (9, 10). Determining the distribution of HPV genotypes in Yueyang city will aid in cervical screening and vaccination and provide a scientific basis for the prevention and treatment of cervical cancer in the city.

Therefore, this study aimed to investigate the epidemiological characteristics of HPV genotypes in women from Yueyang city.

## Materials and methods

### Research subjects

In this cross-sectional study, participants were 125,604 women who underwent HPV typing tests at eight hospitals in Yueyang city from September 2019 to September 2022 (Yueyang People’s Hospital, Yueyang Central Hospital, Miluo People’s Hospital, Huarong County People’s Hospital, Pingjiang County People’s Hospital, Pingjiang County Maternal and Child Health Hospital, Yueyang County People’s Hospital, and Linxiang People’s Hospital). The age range of the participants was from 11 to 97 years, the average age is 43.42 ± 11.078 years old. The sampling criteria were as follows: non-menstrual period, no sexual activity within 24 h, no vaginal medication within 3 days, no vaginal irrigation, and no prior application of acetic acid or iodine solution. This study was approved by the ethics committee of the hospitals mentioned above.

This study focused on HPV infection prevalence as the main research index, which is known to be 15.5% based on the results of similar published literature (11). Using the following formula, α = 0.05 (two-sided test) was selected, the allowable error δ = ±0.22%, 𝑍1-𝛼/2 = 1.96, and *p* = 15.5%, 𝑛= (𝑍1- 𝛼/2𝛿)2𝑝(1-𝑝) calculated with HPV infection prevalence yields 103,958 cases to be investigated. Considering that the rate of lost visits is 10 to 20%, the study included a total of 125,604 people, which can ensure the accuracy and scientific validity of the results of the study.

### HPV testing

The HPV genotyping test kits applied by Yueyang People’s Hospital, Yueyang Central Hospital, Miluo People’s Hospital, Miluo Maternal and Child Health Hospital were provided by 21 HPV GenoArray Diagnostic Kit (HBGA-21PKG) (Hybribio, China, Guangdong), the HPV test kits applied by Pingjiang People’s Hospital, Pingjiang Maternal and Child Health Hospital, Hualong People’s Hospital were provided by 23 HPV Genotyping Panel kit from (CFDA: 20193401918) (Yanengbio, China, Shenzhen), and the HPV test kits applied by Linxiang People’s Hospital were provided by 26 HPV Genotyping Panel kit from (CFDA: 20173404697) (TELLGEN Life Science, China, Shanghai). The sensitivity and specificity of these kits were higher than 95% compared to FDA approved kit.

In this study, 21 HPV genotypes were detected: 15 HR-HPV genotypes (16, 18, 31, 33, 35, 39, 45, 51, 52, 53, 56, 58, 59, 66, and 68) and 6 LR-HPV genotypes (6, 11, 42, 43, 44, and 81). Testing for the different HPV genotypes was conducted to identify the distribution and genotype of HR-HPV and low-risk HPV (LR-HPV) subtypes. Positive and negative controls were used for each test, and the test kit instructions were strictly followed.

### Statistical analysis

SPSS Statistics 23.0 software was used for data analysis. Categorical variables were expressed as frequencies (proportions). Categorical variables were compared using Chi-square or Fisher’s exact probability tests. Pairwise comparison between groups was performed using the Bonferroni method, adjusting for the level of *p*-values. *p*-values <0.05 (two-sided) were considered statistically significant.

## Results

### Prevalence of HPV detection and subtype distribution

**Figure 1:**
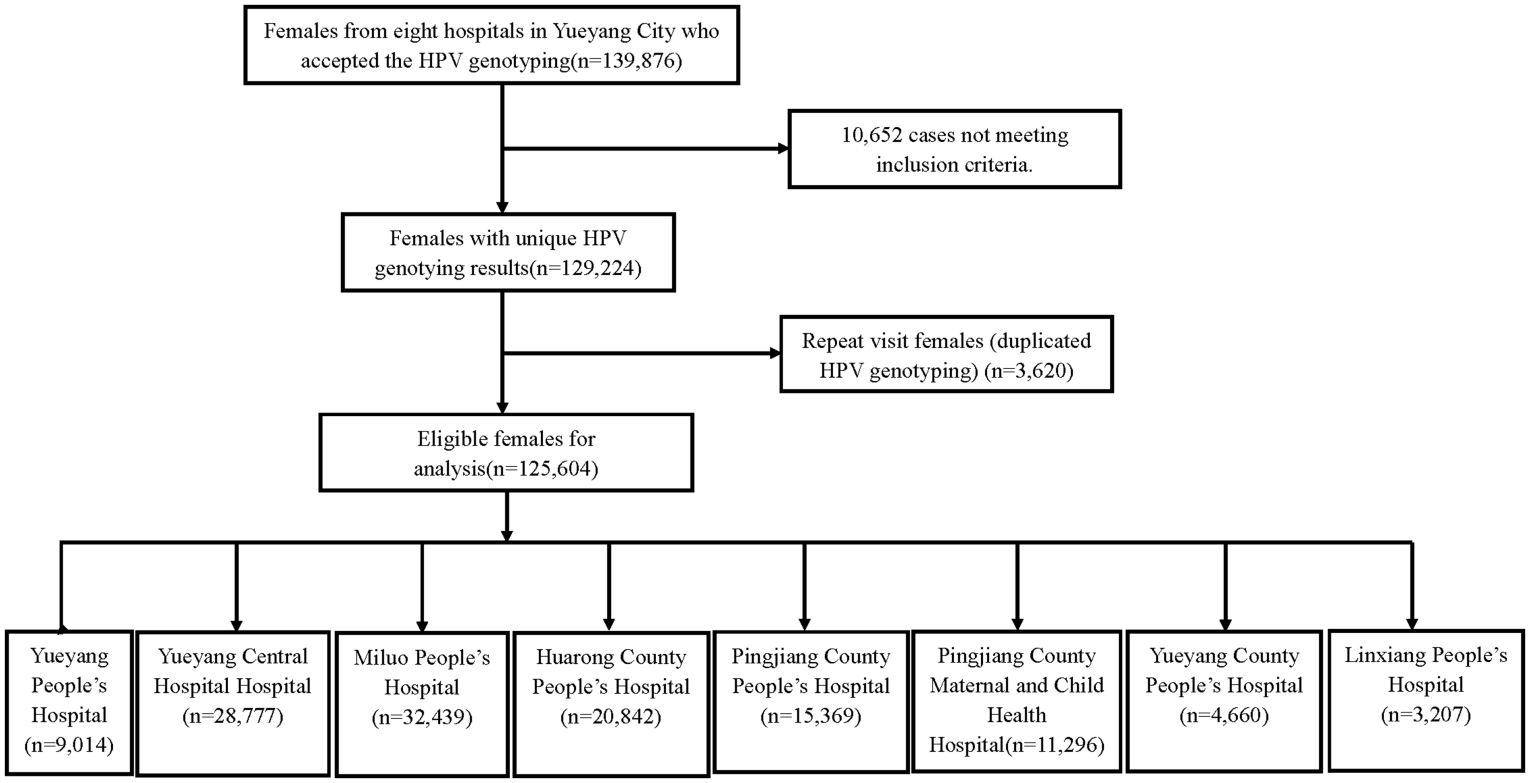
Study population.

In this study, 125,604 women from eight hospitals in Yueyang city met the selection criteria (Figure 1). The age of the participants was from 11 to 97 years, and the average age was 43.42 ± 11.078 years. Among these participants, 25,714 showed positive results for HPV, and the prevalence of HPV detection was 20.5% (95%CI: 20.2–20.7%). Overall, 21,946 HR-HPV cases were noted, and the prevalence of HR-HPV and LR-HPV was 17.5% (95%CI: 17.3–17.7%) and 5.0% (95%CI: 4.9–5.1%), respectively.

### Infection and distribution characteristics of different HPV genotypes

**Figure 2:**
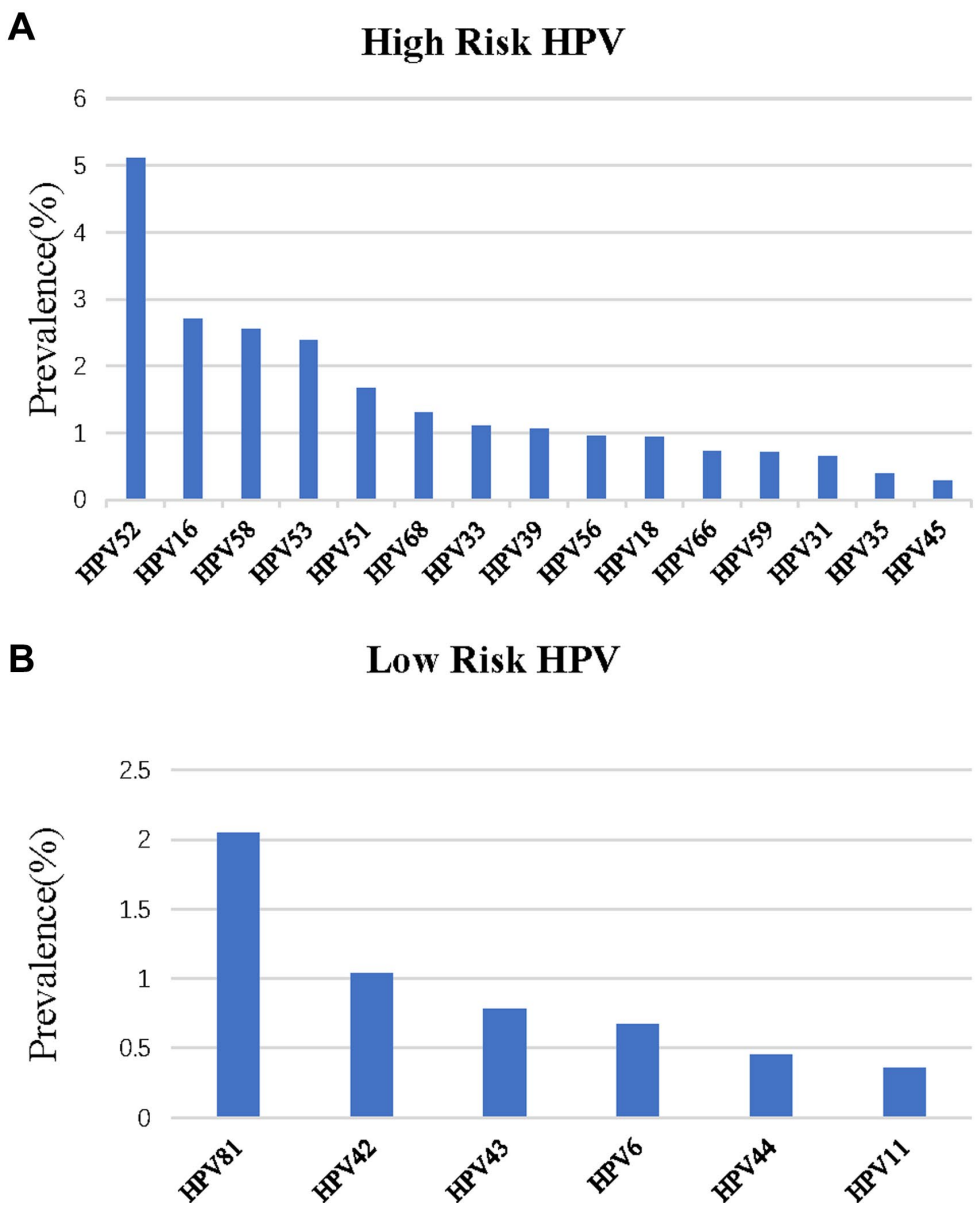
Distribution of HPV genotypes among samples: **(A)** prevalence of High-Risk (HR) HPV among samples: **(B)** prevalence of Low -Risk (LR) HPV among samples.

The top five subtypes of HR-HPV detected in order of prevalence (from the highest to the lowest) were as follows: HPV 52 (6,413 cases, OR = 5.1%; 95%CI, 5.0–5.2%), HPV 16 (3,410 cases, OR = 2.7%; 95%CI, 2.6–2.8%), HPV 58 (3,208 cases, OR = 2.6%; 95%CI, 2.5–2.6%), HPV 53 (3,004 cases, OR = 2.4%; 95%CI, 2.3–2.5%), and HPV 51 (2,093 cases, OR = 1.7%; 95%CI, 1.6–1.7%). The top three subtypes of LR-HPV (from the highest to the lowest) were as follows: HPV 81 (2,576 cases, OR = 2.1%; 95%CI, 2.0–2.1%), HPV 42 (1,303 cases, OR = 1.0%; 95%CI, 1.0–1.1%), and HPV 43 (978 cases, OR = 0.8%; 95%CI, 0.7–0.8%) (Table 1; Figure 2). Single subtype infections were the most common and accounted for 19,293 cases or 15.3% (95%CI: 15.2–15.6%) of the group. Dual subtype infections accounted for 4,673 cases or 3.7% (95%CI: 3.6–3.8%). Multiple infections (≥3) accounted for 1,957 cases or 1.6% (95%CI, 1.5–1.6%) (*p <* 0.001, Table 2).

**Table 1:** Single and multiple type infection rates different HPV subtypes in Yueyang.

HPV subtype	Positive, *n* (%)	95%CI for positive (%)	Single-type infection, *n* (%)	Multiple-type infection, *n* (%)
HPV52	6,413 (5.11)	4.98–5.23	4,054 (3.23)	2,359 (1.88)
HPV16	3,410 (2.71)	2.63–2.80	2,049 (1.63)	1,361 (1.08)
HPV58	3,208 (2.55)	2.47–2.64	1,824 (1.45)	1,384 (1.10)
HPV53	3,004 (2.39)	2.31–2.48	1,626 (1.29)	1,378 (1.10)
HPV81	2,576 (2.05)	1.97–2.13	1,343 (1.07)	1,233 (0.98)
HPV51	2,093 (1.67)	1.60–1.74	1,044 (0.83)	1,049 (0.84)
HPV68	1,645 (1.31)	1.25–1.37	826 (0.66)	819 (0.65)
HPV33	1,397 (1.11)	1.05–1.17	746 (0.59)	651 (0.52)
HPV39	1,334 (1.06)	1.01–1.12	694 (0.55)	640 (0.51)
HPV42	1,303 (1.04)	0.98–1.09	623 (0.50)	680 (0.54)
HPV56	1,210 (0.96)	0.91–1.02	579 (0.46)	631 (0.50)
HPV18	1,177 (0.94)	0.88–0.99	624 (0.50)	553 (0.44)
HPV43	978 (0.78)	0.73–0.83	450 (0.36)	528 (0.42)
HPV66	915 (0.73)	0.68–0.78	437 (0.35)	478 (0.38)
HPV59	895 (0.71)	0.67–0.76	415 (0.33)	480 (0.38)
HPV31	815 (0.65)	0.60–0.69	391 (0.31)	424 (0.34)
HPV6	840 (0.67)	0.62–0.71	399 (0.32)	441 (0.35)
HPV44	569 (0.45)	0.42–0.49	371 (0.30)	198 (0.16)
HPV35	502 (0.40)	0.36–0.43	208 (0.17)	294 (0.23)
HPV11	451 (0.36)	0.33–0.39	216 (0.17)	235 (0.19)
HPV45	360 (0.29)	0.26–0.32	162 (0.13)	198 (0.16)

**Table 2:** Prevalence of single and multiple HPV infection (*N* = 25,833).

Genotype of HPV infection	Number of cases	Prevalence, %
1 HPV subtype^a^	19,203	15.29
2 HPV subtype	4,673	3.72
3 HPV subtype	1,364	1.09
4 HPV subtype	401	0.32
5 HPV subtype	119	0.09
6 HPV subtype	41	0.03
7 HPV subtype	20	0.02
8 HPV subtype	7	0.01
9 HPV subtype	5	0.004

a Higher prevalence of single than multiple HPV infections. *p* < 0.001.

### HPV infections in women across different age groups

Table 3 shows the prevalence of HPV infections in different age groups. Of the 125,604 testing samples, they were classified into six age groups as follows: <25 years, 25–34 years, 35–44 years, 45–54 years, 55–64 years, and ≥ 65 years. The ≥65-year-old group had the highest prevalence of 32.3% (95%CI: 30.9–33.6%) (*p* < 0.001).

**Table 3:** Prevalence of HPV infection by age group (*N* = 125,604).

Prevalence of HPV infection (*n*/%)							
HPV genotype	<25 (*n* = 3,343)	25–34 (*n* = 27,695)	35–44 (*n* = 34,346)	45–54 (*n* = 41,748)	55–64 (*n* = 13,863)	≥65 (*n* = 4,609)	*p* value^a^
Any HPV Infection^b^	892 (26.68)	4,735 (17.10)	6,055 (17.63)	8,519 (20.41)	4,025 (29.03)	1,488 (32.28)	<0.001
High-risk HPV							
Any high-risk HPV^b^	583 (17.44)	4,068 (14.69)	5,176 (15.07)	7,235 (17.33)	3,520 (25.39)	1,364 (29.59)	<0.001
52^b^	194 (5.80)	1,285 (4.64)	1,499 (4.36)	2,075 (4.97)	1,019 (7.35)	389 (8.44)	<0.001
16^b^	170 (5.09)	683 (2.47)	742 (2.16)	1,041 (2.49)	524 (3.78)	275 (5.97)	<0.001
58^b^	93 (2.78)	576 (2.08)	666 (1.94)	1,039 (2.49)	582 (4.20)	276 (5.99)	<0.001
53^b^	80 (2.39)	475 (1.72)	643 (1.87)	1,051 (2.52)	556 (4.01)	207 (4.49)	<0.001
51	96 (2.87)	430 (1.55)	471 (1.37)	633 (1.52)	331 (2.39)	145 (3.15)	-
68	51 (1.53)	297 (1.07)	381 (1.11)	524 (1.26)	292 (2.11)	111 (2.41)	-
33	62 (1.85)	201 (0.73)	288 (0.84)	425 (1.02)	277 (2.00)	151 (3.28)	-
39	45 (1.35)	251 (0.91)	319 (0.93)	402 (0.96)	202 (1.46)	105 (2.28)	-
56	51 (1.53)	203 (0.73)	224 (0.65)	392 (0.94)	248 (1.79)	106 (2.30)	-
18	48 (1.44)	221 (0.80)	290 (0.84)	365 (0.87)	186 (1.34)	73 (1.58)	-
66	39 (1.17)	160 (0.58)	213 (0.62)	281 (0.67)	164 (1.18)	66 (1.43)	-
59	49 (1.47)	182 (0.66)	192 (0.56)	257 (0.62)	151 (1.09)	68 (1.48)	-
31	27 (0.81)	142 (0.51)	183 (0.53)	235 (0.56)	151 (1.09)	87 (1.89)	-
35	14 (0.42)	84 (0.30)	105 (0.31)	156 (0.37)	103 (0.74)	44 (0.95)	-
45	17 (0.51)	60 (0.22)	64 (0.19)	133 (0.32)	58 (0.42)	30 (0.65)	-
Low-risk HPV							
Any low-risk HPV^b^	269 (8.05)	1,060 (3.83)	1,318 (3.84)	2,118 (5.07)	1,099 (7.93)	382 (8.29)	<0.001
81^b^	87 (2.60)	383 (1.38)	538 (1.57)	935 (2.24)	495 (3.57)	149 (3.23)	<0.001
42	55 (1.65)	222 (0.80)	246 (0.72)	404 (0.97)	261 (1.88)	125 (2.71)	-
43	55 (1.65)	169 (0.61)	196 (0.57)	355 (0.85)	152 (1.10)	59 (1.28)	-
6	58 (1.73)	181 (0.65)	186 (0.54)	223 (0.53)	133 (0.96)	55 (1.19)	-
44	9 (0.27)	81 (0.29)	148 (0.43)	208 (0.50)	92 (0.66)	32 (0.69)	-
11	50 (1.50)	89 (0.32)	86 (0.25)	134 (0.32)	70 (0.50)	25 (0.54)	-
1 HPV infection^b^	599 (17.92)	3,659 (13.21)	4,834 (14.07)	6,470 (15.50)	2,654 (19.14)	867 (18.81)	-
2 HPV infection^b^	183 (5.47)	816 (2.95)	926 (2.70)	1,510 (3.62)	902 (6.15)	337 (7.31)	-
Multiple HPV infection^b^	110 (3.29)	260 (0.94)	295 (0.86)	539 (1.29)	469 (3.38)	284 (6.16)	-

a Overall differences in the prevalence of HPV infection between age groups were analyzed using the Chi-square test.

b Pairwise comparisons of HPV prevalence in different age groups were performed using the Bonferroni correction.

**Figure 3:**
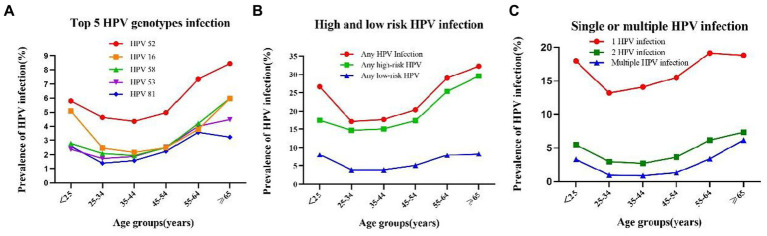
Prevalence and of HPV grouped by age: **(A)** Prevalence and of HPV 52,26,58,53 and 81 in each age group: **(B)** prevalence of High-Risk (HR), Low -Risk (LR), and HPV infection in each age group; **(C)** Prevalence and single and multiple infection of HPV in each age group.

There were two peaks in HPV prevalence. The first peak was for women aged <25 (OR = 26.7%; 95%CI, 25.2–28.2%) and the second was for women aged 55 and over (OR = 29.9%; 95%CI, 29.2–30.5%) (Bonferroni correction, *p* < 0.001) (Table 3, Figure 3). Any HPV infections and HR-HPV infections also exhibited these two peaks in the same age groups (Bonferroni correction, *p* < 0.001, respectively) (Table 3, Figure 3B), same trend for double HPV infection, and multiple HPV infection groups (Figure 3C). These two HPV prevalence peaks were also observed in the prevalence of HPV52, HPV16, and HPV58 infection (Bonferroni correction, *p* < 0.001, respectively) (Table 3, Figure 3A).

However, when we compared the prevalence of LR-HPV infection, single HPV infection, HPV53 and HPV81 infection in different age groups, the second peak in the group aged 55–64 (Table 3, Figure 3) (Bonferroni correction, *p* < 0.001, respectively).

Among the 21 HPV subtypes, the prevalence of HPV 52 subtype was the highest in all the age groups in addition to the ≥65 years old group, with the prevalence of 8.4%(95%CI: 7.6–9.2%) (*p* < 0.001). Of the six LR-HPV subtypes, HPV 81 had the highest prevalence (*p* < 0.001). The subtype prevalence differed for each age group. For the two age groups with larger sample sizes, namely the 35–44-year-old group (n = 34,346) and the 45–54-year-old group (*n* = 41,748), the top five subtypes were HPV 52, HPV 16, HPV 58, HPV 53, and HPV 81 and HPV 52, HPV 53, HPV 16, HPV 58, and HPV 81, respectively. For the <25-year-old group (*n* = 3,343), which had the fewest number of cases, the top five subtypes were HPV 52, HPV 16, HPV 51, HPV 58, and HPV 81 (Table 3; Figure 3A).

## Discussion

The primary objective of this study was to investigate the prevalence and genotype distribution of HPV infections in Yueyang city. The results of this study showed that the total HPV the prevalence in this region was 20.47%, which was higher than that reported in previous studies in other parts of China, such as Huzhou (15.5%), Tianjin (14.71%), Xinjiang (14.0%), and Shanghai (17.92 and 18.98%) (8, 11–14). However, the HPV prevalence in Yueyang was lower than that in Shandong (28.4%), Changsha (26.5%), Chongqing (26.2%), and Beijing (21.06%) (14–17). This indicates that the HPV prevalence differ according to regions. In the present study, the prevalence of HR-HPV was 17.47%, which was higher than that of LR-HPV (4.97%). This indicates that the Yueyang region is dominated by HR-HPV infection, an observation that is consistent with the findings of most previous studies in China (17–20). Persistent HR-HPV infection can cause cervical abnormalities and lead to cervical cancer (21, 22). Therefore, there is an urgent need to enhance HPV infection screening and genotype monitoring in Yueyang city.

Epidemiological studies have shown that the five most common genotypes worldwide are HPV 16, HPV 18, HPV 52, HPV 31, and HPV 58 (23) and that the most common genotypes in Asia are HPV 16, HPV 52, HPV 58, HPV 18, and HPV 56 (24). The present study showed that HPV 52 (5.11%), HPV 16 (2.71%), HPV 58 (2.55%), HPV 53 (2.39%), and HPV 81 (2.05%) are the dominant subtypes in Yueyang, which are the same as those reported by Yurong Zhu in Huzhou (11), but different from those dominant in Zhejiang (52, 58, 16, 51), Xinjiang A(16, 52, 58, 53, 31), and Shanghai (16, 58, 52, 51, 54) (8, 14, 25). This means that the prevalence of different HPV subtypes differs depending on the region, socioeconomic levels, resource availability, and ethnicity.

In the present study, the most common HPV genotype was HPV 52. This finding is the same as that in Beijing, Jiangsu, Zhejiang, and Guangdong (17, 25–27). Many studies have also concluded that HPV 16 is the most common genotype, as seen in Xinjiang, Tianjin, Shanghai, and Shandong (8, 12, 14, 28). That said, bivalent vaccines, which can prevent HPV 16 and HPV 18 infections, and quadrivalent vaccines, which can prevent HPV 6, HPV 11, HPV 16, and HPV 18 infections, do not prevent HPV 52 and HPV 58 infections. Currently, only 9-valent vaccines can prevent HPV 52, HPV 16, and HPV 58 infections, which have high prevalence in Yueyang, as well as prevent HPV 6, HPV 11, HPV 18, HPV 31, HPV 33, and HPV 45 infections. However, women who have received 9-valent vaccinations are a minority in Yueyang. Therefore, it is critical to enhance efforts in increasing vaccination rates among women in Yueyang. In addition, the present study found that HPV 53 and HPV 81 are relatively more prevalent in Yueyang. These two subtypes are not covered by the 9-valent vaccines; therefore, more research is required to determine whether it is necessary to develop and distribute vaccines in Yueyang to target HPV 53 and 81 specifically.

Numerous studies have shown that HPV infection is significantly age-specific, The data in the present study supports previous findings of a bimodal distribution of the HPV prevalence in China (11, 19, 29–31), as our results showed incidence peaks for women aged <25 and ≥ 55 years. The incidence peak for young women aged <25 years could perhaps be attributed to their insufficient understanding of sex and their immature immune systems. For women aged ≥55 years, they may have been affected by physiological and immune disorders during menopausal transition. Therefore, we recommend enhancing HPV vaccination programs and lowering the age of cervical cancer screening for young women in Yueyang.

This study has some limitations. First, since the screening samples were taken from various hospitals, we cannot rule out operational mistakes and errors in the results. Second, the sample also included patients who actively sought HPV testing because they were experiencing symptoms, which may have led to higher rates of HPV infection. Finally, because not all women had the opportunity to undergo free cervical cancer screening, data from women with a lower socioeconomic status and cervical cancer awareness would have been left out from the study. Hence, such data could not be deemed as fully representative of the women in Yueyang. We recommend that enhancing efforts to promote and increase cervical cancer screening and to make free screenings available to more women should be continued, which will increase the quantity and comprehensiveness of data for future research.

In conclusion, the data from this multi-center study have shown that the HPV prevalence of women in Yueyang was 20.47%. HR-HPV was the dominant subtype, and the five most common genotypes were HPV 52, HPV 16, HPV 58, HPV 53, and HPV 81. Based on age groups, incidence peaks were observed for women aged <25 years, 55–64 years, and ≥ 65 years. We believe that this study will provide significant value to future cervical cancer screening and HPV vaccine development strategies in Yueyang.

## Data availability statement

The original contributions presented in the study are included in the article/supplementary material, further inquiries can be directed to the corresponding authors.

## Ethics statement

This study scheme was reviewed and approved by the Ethics Committee of Yueyang People’s Hospital, approval number: 2019003. The patients/participants provided their written informed consent to participate in this study. The research was conducted ethically in accordance with the World Medical Association Declaration of Helsinki.

## Author contributions

YG and XZ conceived and designed the study and took responsibility for the content of the manuscript. JH and MZ analyzed the data, took responsibility for the accuracy of the data analysis, and wrote the first draft of the manuscript. YG and XZ reviewed the paper. CL, BX, YL, LW, LZ, ML, and ZZ were responsible for clinical data collection. All authors contributed to the article and approved the submitted version.

## Funding

The work was supported by the Health Commission of Hunan Province (number: 20201355).

## Conflict of interest

The authors declare that the research was conducted in the absence of any commercial or financial relationships that could be construed as a potential conflict of interest.

## Publisher’s note

All claims expressed in this article are solely those of the authors and do not necessarily represent those of their affiliated organizations, or those of the publisher, the editors and the reviewers. Any product that may be evaluated in this article, or claim that may be made by its manufacturer, is not guaranteed or endorsed by the publisher.
